# Ventilation and Pollutant Concentration for the Pedestrian Zone, the Near-Wall Zone, and the Canopy Layer at Urban Intersections

**DOI:** 10.3390/ijerph182111080

**Published:** 2021-10-21

**Authors:** Mingjie Zhang, Zhi Gao, Xin Guo, Jialei Shen

**Affiliations:** 1 School of Architecture and Urban Planning, Nanjing University, 22 Hankou Road, Nanjing 210093, China; mzhang@smail.nju.edu.cn (M.Z.); xinguo@smail.nju.edu.cn (X.G.); 2 Joint International Research Laboratory of Eco-Urban Design, Tongji University, Ministry of Education, 1239 Siping Road, Shanghai 200092, China; 3 Department of Mechanical and Aerospace Engineering, Syracuse University, Syracuse, NY 13210, USA; jshen20@syr.edu

**Keywords:** intersections typology, CFD simulations, pedestrian ventilation, near-wall concentration, volumetric flow rate, age of air

## Abstract

To gain further insight into the ventilation at urban street intersections, this study conducted 3D simulations of the ventilation at right- and oblique-angled intersections under eight wind directions by using the Reynolds-averaged Navier–Stokes (RANS) κ-ε turbulence model. The divergent responses of ventilation and pollution concentration for the pedestrian zone (ped), the near-wall zone (nwz), and the canopy layer to the change in intersection typology and wind direction were investigated. The flow characteristics of the intersections, taken as the air flow hub, were explored by employing indices such as the minimum flow ratio (β) between horizontal openings. The results show that oblique wind directions lead to a lower total volumetric flow rate (Q_total_) but a higher β value for right-angled intersections. For T-shaped intersections, a larger cross-sectional area for the outflow helps to increase Q_total_. Oblique-angled intersections, for example, the X-shaped intersection, experience a more significant difference in Q_total_ but a steady value of β when the wind direction changes. The vertical air-exchange rate for the intersection was particularly significant when the wind directions were parallel to the street orientation or when there was no opening in the inflow direction. The spatially averaged normalized pollutant concentration and age of air ($\bar{\tau^{*}}$) for the pedestrian zone and the canopy layer showed similar changing trends for most of the cases, while in some cases, only the $\bar{\tau_{\mathrm{ped}}^{*}}$ or $\bar{\tau_{\mathrm{nwz}}^{*}}$ changed obviously. These findings reveal the impact mechanism of intersection configuration on urban local ventilation and pollutant diffusion.

## 1. Introduction

In a realistic urban context, street canyons and adjacent intersections are commonly considered as two basic physical elements with a symbiotic relationship [1,2]. An intersection connects three or more street segments in different directions, while two intersections determine a street canyon along the long axis. Multiple traffic flows from the street, including pedestrians, cyclists, and motor vehicles, converge at intersections. Traffic flows slow or stop at intersections before moving on to their destination streets. Therefore, intersection typologies are primarily shaped by street networks [3], particularly the number of branches, the angle between street segments, and the dimensions of each adjacent street.

To improve the traffic flow for expressways and highways, grading separation is commonly adopted by setting up roads with different elevations. Grade crossings, that is, crossings intersecting at a plane, are more common in downtown areas, connecting primary and secondary roads. According to the number of branches, grade crossings can be categorized into three-way, four-way, five-way, or higher. In grid-plan urban areas, east–west oriented streets are perpendicular to north–south oriented streets, forming regular-shaped intersections [4]. Regular intersections evolve into oblique intersections when encountering radial or irregular street grids. To smooth the traffic flow, a traffic island set up at the intersection center (i.e., roundabouts) is used to distribute traffic flows instead of traffic signals [5]. When using roundabouts, traffic travels around a central island, which is considered more effective and suitable for junctions where higher-intensity traffic flow occurs.

From the perspective of the urban canyon environment, intersections are regarded as a lateral opening for street canyons, which provide an essential horizontal flow exchange other than the vertical air exchange at the top of the canyon [6]. The ventilation for the intersection itself, such as the flow pattern, vortices, wind velocity ratio, vertical distribution, and air flow rate, further affects the ventilation within nearby street segments [7]. Previous studies have reported the benefits of intersections in enhancing ventilation in street canyons by bringing more air flow into their centers [8,9]. Thus, reasonable intersection density can help improve urban local ventilation and reduce pollutant concentration, in addition to strengthening the flexibility of urban street networks and the links between urban areas.

One of the concerns within intersections and street canyons is pedestrians. Pedestrians encounter the bottom flow from the ground to 2 m, which carries pollutants released by motor vehicles [10]. The street ends and intersections are noticeably more complex than the two-dimensional middle slice of the canyon. However, chamfering [11], setback [12], and disjointing [13] of buildings at street corners may help enlarge the space to a certain extent. Thus, more sophisticated flow form at street ends and intersections, as evidenced by three-dimensional vortices and the complex stratification of velocity. Another concern is the street-facing building spaces. Building facades serve as the lateral boundary of a street canyon or an intersection [14]. The underlying risks include the inward transport of traffic-related pollutants through infiltration, natural ventilation, or even mechanical air systems, which subsequently cause the deterioration of indoor air quality [14,15,16]. Under specific conditions, the top floor of a building may even face a higher ambient pollutant concentration under specific conditions owing to the vertical diffusion and transport of pollutants [15].

Although not a hotspot, the ventilation and pollutant dispersion at or near street intersections and their contributing factors were quantitatively investigated in previous studies. Some researchers have used field measurements to explore the spatiotemporal distribution of wind velocity and pollutant concentration at intersections [16]. The dynamic traffic flow and time-varying local wind velocity showed considerable influence; however, the impact of factors such as building morphology, intersection configuration, and prevailing wind directions cannot be accessed without parametric studies. Alternatively, ventilation at or near intersections has been analyzed using wind tunnels [9,17] and computational fluid dynamics (CFD) methods [8,18]. In more recent studies, intersections were modeled as a node with certain resistance of the urban street network model for ventilation or pollutant dispersion analysis [6].

Ahmad et al. [17] reviewed studies on the investigation of the air flow and pollutant concentration for urban street canyons and intersections and found that a significant street-level flow exchange existed at the intersections, which could help change the street canyon vortices into helical or intermittent ones. Some wind tunnel studies [19,20] have explored pollutant concentrations at regular intersections within uniform rectangular urban blocks under eight wind directions. Significant concentration variations throughout the intersection were observed at the pedestrian level, and the parallel and perpendicular winds to the long street led to street-level minimum and maximum concentrations, respectively. Wang and McNamara [7] investigated the dispersion at urban intersections under wind direction not aligned with the upwind street and found that the in-canyon spiraling flow led to ground-level emissions being elevated to higher altitudes and crosswind streets. Yassin et al. [8] reported that the intersection configuration, such as regular-, T-, and skew-shaped, impacts air flow and pollutant dispersion within urban canyons based on the simulation of the horizontal and vertical distribution of wind velocity and pollutant concentration. Shen et al. [11] assessed pedestrian and street canyon ventilation using indices such as net escape velocity and age of air for six irregular real-world cases under the wind direction parallel to the street and concluded that octagon and oblique intersections were favorable for central street ventilation. He et al. [12] conducted a numerical parametric study on pedestrian ventilation at four-way street intersections of 13 angular patterns within a high-rise urban area, confirming the effect of both the prevailing wind direction and the relative orientation of adjacent street segments and concluded that moderate angles for downstream segments help balance the downstream and lateral flow penetrations. Guo et al. [21] simulated pollutant dispersion and ventilation, particularly at street and pedestrian levels, by normalized concentration and net escape velocity, at three types of intersections with different green configurations, and reported that the presence of greening could worsen the pollutant conditions at intersections to different extents because of the change in intersection typologies and approaching wind directions.

Although the ventilation and air quality at or near intersections have been investigated from multiple perspectives in previous studies, the divergent response of ventilation for pedestrian-level, near-wall zone, and canopy layer to the change of intersection typologies and approaching wind directions are still lacking. Thus, this study aims to provide a better insight into the effects of different types of intersections within urban-like geometries, considering contributing factors such as the number of branches, angles between street segments, and intersection shapes. Numerical simulations of ventilation for the intersections were conducted under eight wind directions shifting in a clockwise manner at intervals of 45°. The results of different cases were analyzed by employing the indices usually used for urban areas and building ventilation, for example, the volumetric flow rate and age of air. It is expected that this work will help to predict the ventilation for pedestrian and street-facing buildings, which further contributes to improving the ventilation by optimizing street intersection typologies.

## 2. Methodology

**Figure 1:**
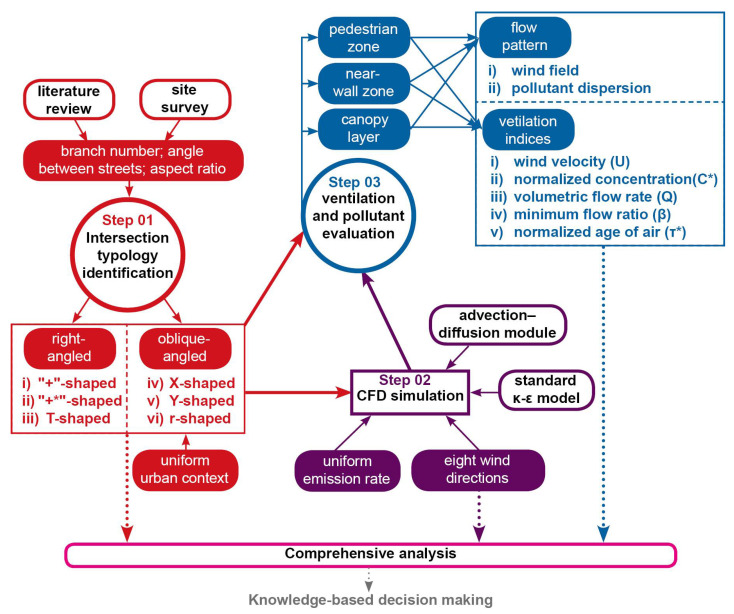
Methodology flowchart showing the major steps in this study: the main aspects are shown in solid shapes, and the methods in wireframed shapes.

An overview of the methodology used in this study is shown in Figure 1. In step 1, the identification of intersection typologies was carried out by summarizing categories of street intersections using morphological factors such as the number of branches and street aspect ratio and establishing research cases (see Section 2.1 for details). In step 2, the flow field and pollution diffusion for the studied cases were simulated using eight approaching wind directions to explore the response of ventilation performance for street intersections to different wind directions (see Section 2.2). In step 3, the ventilation and pollution dispersion for the studied cases were evaluated from the perspectives of pedestrian zone, near-wall zone, and canopy layer by exploring the flow field and by employing ventilation indices; a comparative study was undertaken using dimensionless parameters (see Section 2.3 for details).

### 2.1. Description of the Investigated Cases

**Figure 2:**
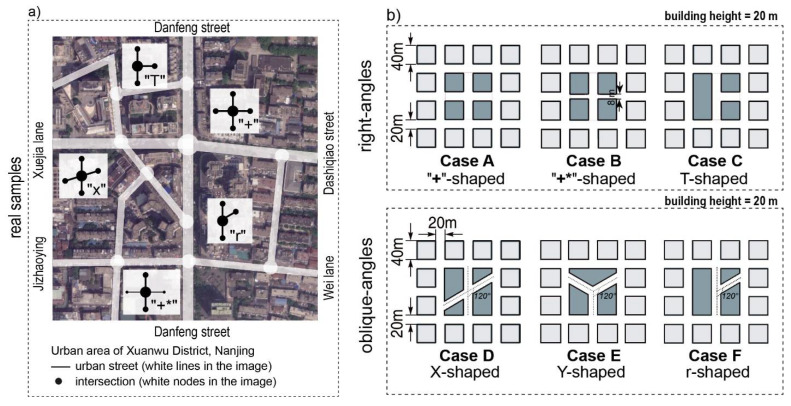
(**a**) Diagram showing several typical intersections in Nanjing, China (base image source: Google Earth): “+”-shaped, “+*”-shaped, X-shaped, T-shaped, and r-shaped; (**b**) six cases of street intersections with a uniform context investigated in this study.

Multiple types of street intersections are usually set up to link street segments and form a flexible urban street network. Figure 2a displays an example of an urban area of Xuanwu District, Nanjing, where there are right-angled intersections, such as the “+”-shaped (or regular-shaped) intersection, “+*”-shaped intersection (regular-shaped intersection with a narrower street), T-shaped intersections, and oblique-angled intersections, such as the X-shaped intersection and r-shaped intersection.

In addition to the above five types of intersections, Y-shaped intersections were found to be common intersections in urban street networks [5]. In this study, these six types of intersections, including right- (90°) and oblique-angled (120°), were studied. Sketches of these intersections are displayed in Figure 2b: right-angled (“+”-shaped, “+*”-shaped, and T-shaped), oblique-angled (X-shaped, Y-shaped, and r-shaped). The six intersections were built by modeling building blocks, thus obtaining the corresponding six cases (Cases A–F), as shown in Figure 2b. The effects of morphology, including intersection type, street corner shape, street aspect ratio, and the number of branches, on flow, pollutant dispersion, and ventilation were analyzed.

It should be noted that only the street intersection at the center was considered as the study area. All the cases are composed of street canyons with an aspect ratio (AR) of 1 aside from Case B, which represents narrow streets with an AR of 2.5. The width of the street was assumed to be 20 m, and the height of building H was 20 m, according to field investigations of street widths in Nanjing [11].

In the analysis, each case was further numbered as follows: “Case i—wind direction” where i represents the intersection types A to F (Figure 2). For example, Case A-0 indicates the “+”-shaped intersection for a wind direction (φ) of 0°. Specifically, the south direction (i.e., from bottom to top in Figure 2) is taken as the 0° wind direction, and a 45° interval (moving counter-clockwise) is considered to evaluate the influence of the wind direction change.

### 2.2. CFD Set-Up

Three-dimensional steady-state isothermal simulations were performed using ANSYS FLUENT (Ansys, PA, USA) 15.0, with the Reynolds-averaged Navier–Stokes (RANS) standard κ-ε turbulence model. The standard κ-ε turbulence model is a two-equation model in which the solution of two separate transport equations allows the turbulent velocity and length scales to be independently determined. It is a semi-empirical model based on model transport equations for the turbulence kinetic energy (κ) and dissipation rate (ε). Large eddy simulations (LES) perform better in predicting turbulence in complex geometries. However, the computational cost of the LES model is several times higher than that of the RANS model, and there are still challenges with its application [22]. Therefore, the RANS approach is still the most common approach for predicting the spatial distribution of mean velocity and concentration fields in urban ventilation [7,8,11,21,23,24].

**Figure 3:**
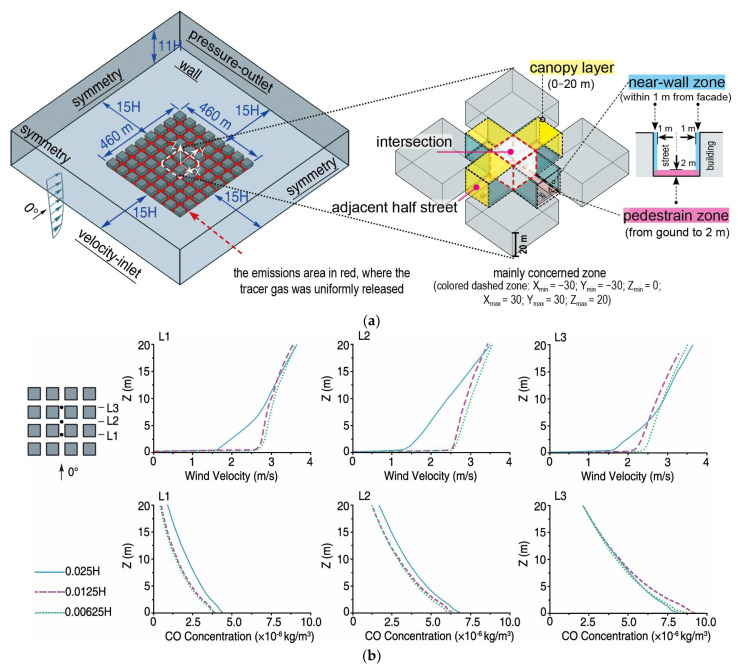
Case A-0 taken as an example. (**a**) Computational domain and boundary conditions used in CFD simulations and (**b**) grid sensitivity study.

The computational domain is shown in Figure 3a. To account for the influence of the surrounding buildings, three arrays of buildings (height H = 20 m) surrounding each case were considered. Considering the change in the wind direction at the entrance, the distance between the lateral boundaries and the urban geometry was set to 15 H, while the domain roof was set to 10 H. The volume of the urban geometry in the computational domain accounted for less than 3% of the calculation area, in accordance with the CFD guidelines [25]. No-slip wall boundary conditions were used for all the solid surfaces. The second-order discretization scheme was used for pressure, and second-order up-winding discretization schemes were used for momentum, k, ɛ, and the scalar to increase accuracy and reduce numerical diffusion. The SIMPLE scheme was used for pressure–velocity coupling. The wind profile at the inlet is [26]

(1) $$\frac{U_{Z}}{U_{H}}={\left(\frac{Z}{H}\right)}^{\alpha }$$

where $U_{Z}$ is the average wind velocity at height Z, U_H_ is the average wind velocity at H, and α = 0.22 is the ground roughness index representing an urban landform with dense buildings. The turbulent kinetic energy $\kappa_{Z}\;$and turbulent dissipation rate $\varepsilon_{Z}\;$profiles are specified as follows:

(2) $$\kappa_{Z}=\frac{u_{*}^{2}}{\sqrt{C_{\mu }}}$$

(3) $$\varepsilon_{Z}=\frac{u_{*}^{3}}{K\left(Z+Z_{0}\right)}\;$$

where $u_{*}\;$is the friction velocity, K=0.4 is the von Karman constant, and $Z_{0}$ is the roughness length. Symmetry boundary conditions were adopted at the lateral sides and top of the computational domain, while a pressure outlet was used at the outlet of the domain, and no-slip wall boundary conditions were used at the ground and building surfaces.

Dispersion calculations were performed using the advection–diffusion module. In turbulent flows, FLUENT computes the mass diffusion as follows:

(4) $$J=-\left(D+\frac{\mu_{t}}{{\mathrm{Sc}}_{t}}\right)\nabla Y$$

where D is the molecular diffusion coefficient for the pollutant in the mixture, Y is the mass fraction of the pollutant, and ρ is the mixture density. Sc_t_ = μ_t_/(ρD_t_) = 0.7 is the turbulent Schmidt number, where D_t_ is the turbulent diffusivity coefficient. The tracer gas (CO, with an emission rate $\;\dot{m}$ = 10^−7^ kg/(m^3^·s)) [24] was uniformly released in the space at the bottom (0–2 m) of the urban geometries (Figure 3a), with the assumption that pollutants released by vehicles are perfectly mixed at ground level due to vehicle motion.

The domain was discretized using a structured grid with hexahedral cells for Cases A, B, and C, while an unstructured grid was employed for the other cases. Case C had the lowest number of cells at 1,978,640. The total number of cells for the other cases was between 3,418,436 and 4,817,223. The area below 2 m at the intersection was refined with a minimum cell size δx = δy = δz = 0.0125 H = 0.25 m. The choice of the grid size was based on a sensitivity test performed using Case A-0 as an example, as shown in Figure 3b. Three lines were selected within the intersection area, and vertical profiles (L1, L2, and L3) were compared for different grid sizes. Wind velocities and concentrations were found to be similar when the grid size refinements were 0.0125 H and 0.00625 H and thus δx = δy = δz = 0.0125 H = 0.25 m was chosen here. The setup employed in this study is similar to several previous studies in terms of the turbulence model, boundary conditions, and grid size [5,11], where the impact of different street morphologies on ventilation within arrays of buildings was studied.

### 2.3. Evaluation Method and Indices

Figure 3a shows the structure of the “+”-shaped intersection (Case A). The area of focus, referred to as “the intersection” in the following discussion, includes the intersection area, and half of each adjacent street canyon that directly interacts with the intersection [27]. The intersection area of Case A has five openings or interfaces: the roof opening to the upper boundary layer and four horizontal openings to the adjacent street canyons [6]. To evaluate the overall ventilation, the volumetric flow rate (Q) at each opening and the total value (Q_total_) were calculated. Considering Case A as an example, the air exchange between the intersection and south street segments (Q_S_) and other adjacent canyons, for example, the east canyon (Q_E_), may differ greatly [9]. Thus, the minimum flow ratio (β) between each horizontal opening was explored to determine whether balanced ventilation existed at the intersection. In addition, the intersection has air exchange with the upper urban boundary layer at the roof opening (Q_roof_), which helps to remove pollutants from the canopy layer [6,23], thus weakening the effect of traffic pollutants on pedestrians and residents. The percentage of Q_roof_ to Q_total_, referred to as λ, was calculated to explore the difference in vertical air-exchange between the cases.

Furthermore, the ventilation and pollutant concentrations in the pedestrian zone, near-wall zone, and canopy layer were analyzed and compared. The pedestrian zone was set from the ground, the entire area of the intersection without considering the difference between the pavement and motor vehicle lane, to a height of 2 m (Figure 3a). For the near-wall zone, the surface effect commonly induces a large velocity gradient near the building wall, which may further affect the local pollutant concentration. Previous studies calculated the data in the zone from the wall to a certain distance away [28,29] as the micro-scale ambient conditions for buildings. The near-wall zone in this study was determined as from the building facades to 1.0 m away (Figure 3a), where the concentrations more directly affect the outdoor pollutant concentration entering the indoor spaces [30]. Meanwhile, the detailed distributions of wind velocity and pollutant concentration were explored based on the results for the pedestrian level, Z = 1.5 m, and the near-wall surface, 0.5 m away from the walls [31,32]. The canopy layer was set from the ground to the roof level, that is, a mean building height of 20 m (Figure 3a).

The studied cases have slightly different release volumes of pollutants that may affect the comparability of the calculated concentration (C, kg/m^3^). Thus, the normalized pollutant concentration ($C^{*}$) was employed to compare the pollutant distribution. The $C^{*}$ value was calculated as follows:

(5) $$C^{*}=\frac{C\times U_{H}\times H^{2}}{\;\dot{m}\;\times V}$$

where $\;\dot{m}$ is the uniform pollutant emission rate (= 10^−7^ kg/(m^3^·s)) and V is the volume of the emission zone where the pollutant is released (m^3^).

The normalized spatial average of pollutant concentration was calculated as follows:

(6) $$\bar{C^{*}}=\overset{}{\underset{\mathrm{Vol}}{\int }}\frac{C^{*}\mathrm{dxdydz}}{\mathrm{Vol}}$$

where Vol is the target control volume (m^3^).

Recent studies have employed ventilation indices to assess urban ventilation based on the assumption that urban areas “inhale” fresh air from upwind and “exhale” air with pollutants downwind. These indices were initially proposed and used to assess indoor ventilation and to evaluate indoor exposure. The local mean age of air (τ) was calculated in this work by adopting the “homogeneous emission method” [24], which was originally proposed for tracer gas techniques. Thus, the pollutants are assumed to be released at a uniform rate within the entire urban area from the space from the ground to 2 m. The method coincides with the worst scenarios in which pollutant generation occurs everywhere within an urban environment. The age of air (τ) can be calculated as:

(7) $$\tau \;=\frac{C}{\;\dot{m}}\;$$

The age of air (τ) was further normalized as τ* in this study to consider the local effective flow rate at the intersection. τ* was calculated as follows:

(8) $$\tau^{*}=\frac{\tau \times Q_{\mathrm{total}}}{V_{\mathrm{cnp}}}$$

where V_cnp_ is the volume (m^3^) of the canopy layer (cnp) at the intersection. Subsequently, the normalized spatial average of the air age was calculated as follows:

(9) $$\bar{\tau^{*}}=\overset{}{\underset{\mathrm{Vol}}{\int }}\frac{\tau^{*}\mathrm{dxdydz}}{\mathrm{Vol}}$$

## 3. Results

### 3.1. Ventilation and Pollutant Concentration at Right-Angled Intersections

**Figure 4:**
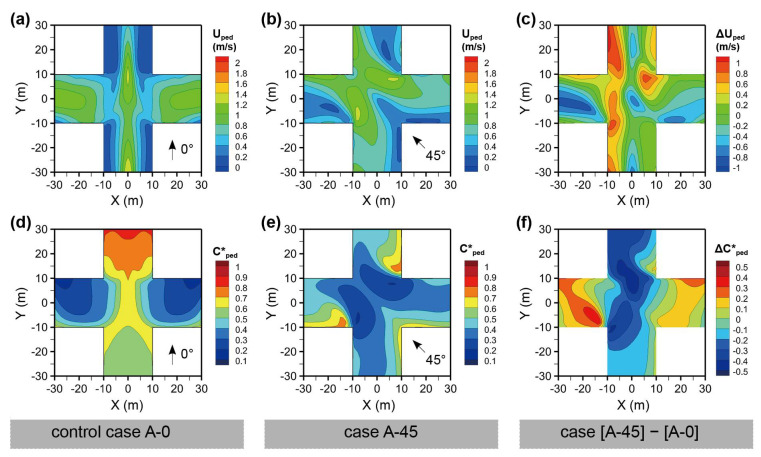
Wind velocity (U) and normalized pollutant concentration ($C_{\mathrm{ped}}^{*}$) at Z = 1.5 m for Case A (regular-shaped intersection); (**a**) $U_{\mathrm{ped}}$ for Cases A-0 and (**b**) A-45; (**c**) Δ$U_{\mathrm{ped}}$ between Cases A-45 and A-0; (**d**) $C_{\mathrm{ped}}^{*}$ for Cases A-0 and (**e**) A-45; (**f**) $C_{\mathrm{ped}}^{*}$ between Cases A-45 and A-0.

Figure 4a,b shows the wind velocity at the pedestrian level ($U_{\mathrm{ped}}$) of the “**+**”- or regular-shaped intersections for the wind directions φ = 0° and 45°, that is, Cases A-0 and A-45. For φ = 0°, the channel effect was observed in the Y-axis street, and a substantially lower $U_{\mathrm{ped}}$ was observed on both the left and right sides. The $U_{\mathrm{ped}}$ on the X-axis street, where there exists a canyon vortex due to the perpendicular wind direction for the street, is more evenly distributed. When φ changes to 45°, oblique to the street orientation, a lower $U_{\mathrm{ped}}$ exists at the leeward sides of the buildings. The spatially averaged $U_{\mathrm{ped}}$ values for the four street segments were reduced. Figure 4c displays the difference between the two cases, with Case A-0 as the control case. It is noted that $U_{\mathrm{ped}}$ increased because of an oblique wind direction, particularly at the windward side of the Y-axis street. Only the leeward side of the left half of the X-axis street experienced a lower wind velocity.

Figure 4d–f shows the normalized pollutant concentrations ($C_{\mathrm{ped}}^{*}$) of Cases A-0 and A-45, and the concentration difference between the two Cases. Case A-0 experiences a higher $C_{\mathrm{ped}}^{*}$ downstream of the Y-axis street owing to the vortex and decreasing trend in wind velocity. The intersection also had a relatively higher $C_{\mathrm{ped}}^{*}$. Case A-45 generally has a lower $C_{\mathrm{ped}}^{*}$, particularly at the intersection and windward sides of the buildings. By comparison, it is noted that the $C_{\mathrm{ped}}^{*}$ was substantially reduced on the Y-axis street, although the $C_{\mathrm{ped}}^{*}$ in areas in the left half of the X-axis street.

**Figure 5:**
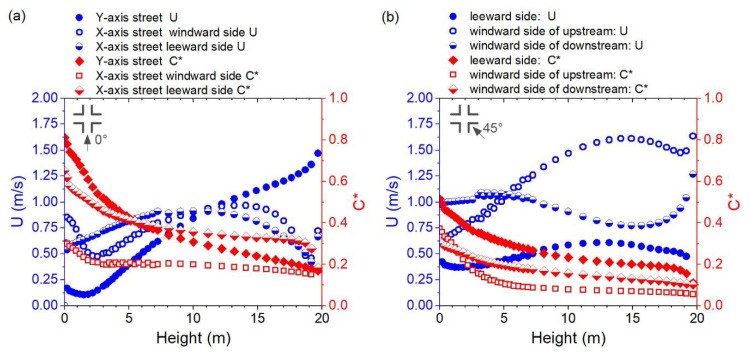
Vertical distribution of wind velocity (U) and normalized pollutant concentration (C*) for the near-wall surfaces (0.5 m away from facades) in Cases (**a**) A-0 and (**b**) A-45.

Figure 5a,b displays the variation in wind velocity ($U_{\mathrm{nwz}}$) and normalized pollutant concentration ($C_{\mathrm{nwz}}^{*}$) along the height (Z = 0–20 m) in the near-wall surface (<0.5 m) for Cases A-0 and A-45. For the Y-axis street in Case A-0, $U_{\mathrm{nwz}}$ shows a general increasing trend above 2 m, while $C_{\mathrm{nwz}}^{*}$ decreases along the height, particularly between 0 and 5 m. The difference in $U_{\mathrm{nwz}}$ for the windward and leeward sides of the X-axis street varied at different height intervals. The windward side has a higher $U_{\mathrm{nwz}}$ below 2 m and above 10 m. However, the $C_{\mathrm{nwz}}^{*}$ on the windward side, where there is more “effective air flow,” was substantially lower. As for Case A-45, the leeward side has an obviously lower $U_{\mathrm{nwz}}$ and higher $C_{\mathrm{nwz}}^{*}$. The windward side of the upstream side has a lower $U_{\mathrm{nwz}}$ at a height below 5 m, while the windward side of the downstream side has a lower $U_{\mathrm{nwz}}$ above 5 m. The windward side of the upstream generally has a lower $C_{\mathrm{nwz}}^{*}$ except for below 2 m. Overall, the $C_{\mathrm{nwz}}^{*}$ below 5 m in Case A-45 was lower than in Case A-0 and the $U_{\mathrm{nwz}}$ at different locations in Case A-45 showed greater divergence than in Case A-0, particularly above 10 m.

**Figure 6:**
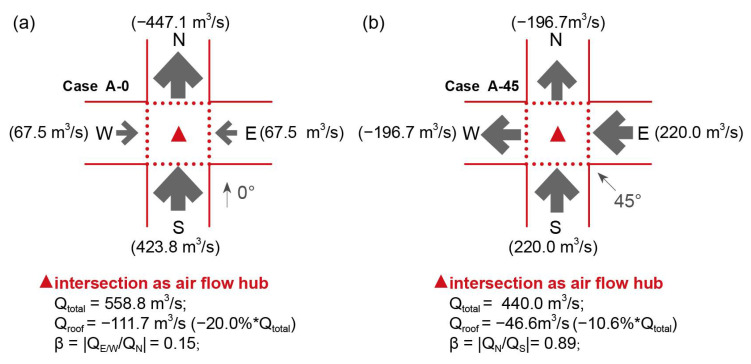
Volumetric flow rate for the intersection and its interfaces in Cases (**a**) A-0 and (**b**) A-45. Positive value indicates inflow; negative value indicates outflow. The factor β refers to the minimum ratio of flow rate between different adjacent street segments.

Figure 6 displays the flow rate for the intersection, which is considered as the air flow hub [9], and its interfaces in Cases A-0 and A-45. When the wind direction φ was 0°, the total volumetric flow rate (Q_total_) of the intersection reached 558.8 m^3^/s. The south interface supplies a large proportion of inflow, in addition to that contributed by the east and west interfaces. The flow ratio (β) of 0.16 indicates an unbalanced ventilation at the intersection, which provides less air exchange for the X-axis street. The outflow mainly passes through the north interface, while there exists still 20.0% of Q_total_ flows through the roof interface to the urban boundary layer. Vertical air-exchange was one of the reasons for the decreasing trend of $C_{\mathrm{nwz}}^{*}$ along the height. When φ changes to 45°, a smaller Q_total_ of 440.0 m^3^/s was observed, but with balanced ventilation for the air exchange between the intersection with adjacent street segments. The south and east interfaces supply an equal amount of inflow, and the north and west interfaces pass through an equal amount of outflow. The vertical air-exchange at the roof interface was approximately 10.6% of Q_total_, which was significantly less than that in Case A-0.

**Figure 7:**
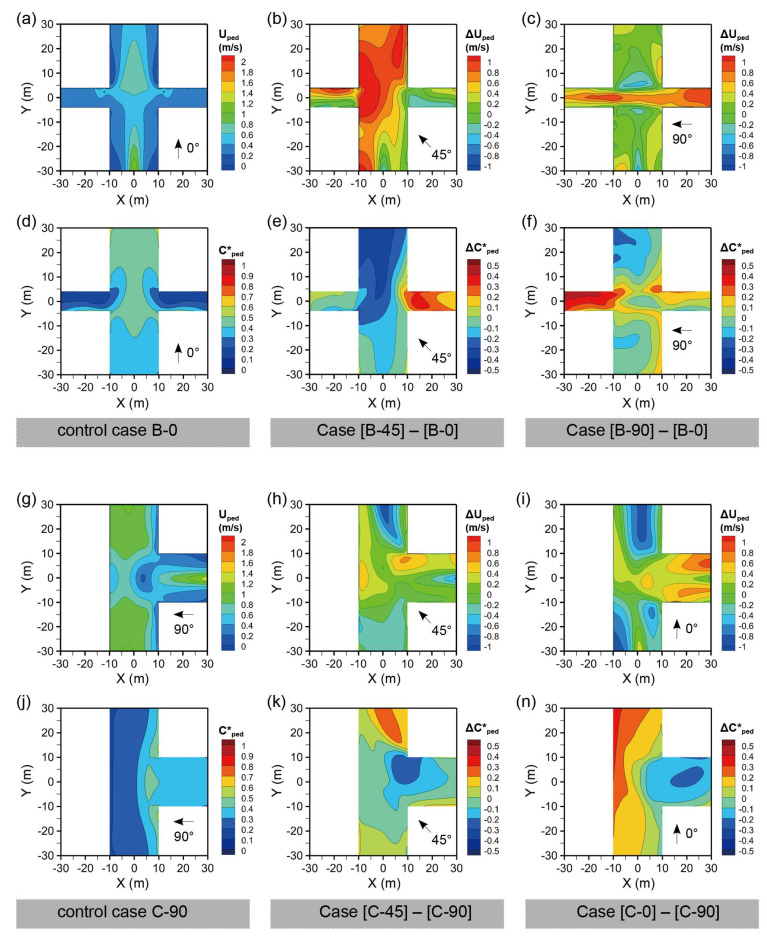
Wind velocity ($U_{\mathrm{ped}}$) and normalized pollutant concentration ($C_{\mathrm{ped}}^{*}$) at Z = 1.5 m, for (**a**,**d**) Case B-0, Δ$U_{\mathrm{ped}}$ and Δ$C_{\mathrm{ped}}^{*}$, and (**b**,**e**) Cases B-45 and (**c**,**f**) B-90, with Case B-0 taken as the control case. $U_{\mathrm{ped}}$ and $C_{\mathrm{ped}}^{*}$ for (**g**,**j**) Case C-90, and Δ$U_{\mathrm{ped}}$ and Δ$C_{\mathrm{ped}}^{*}$ for Cases (**h**,**k**) C-45 and (**i**,**n**) C-90, with Case C-90 taken as the control case.

Figure 7a,d displays the $U_{\mathrm{ped}}$ and $C_{\mathrm{ped}}^{*}$ for the “+*”-shaped intersection, with a narrow X-axis street, for the wind directions φ = 0°, that is, Case B-0. The symmetric wind field with a higher $U_{\mathrm{ped}}$ near the central line of the Y-axis street indicates the channel effect. The X-axis street has a similar $U_{\mathrm{ped}}$ with both sides of the upstream of the Y-axis street, which reflects the poor ventilation in streets with a larger AR (2.25) when the wind direction is perpendicular to the street orientation. A relatively higher $C_{\mathrm{ped}}^{*}$ was observed downstream of the Y-axis street due to pollutant accumulation. The axis street has a lower $C_{\mathrm{ped}}^{*}$ except for the area near the leeward walls. Figure 7b,e shows the difference between $U_{\mathrm{ped}}$ and $C_{\mathrm{ped}}^{*}$ when φ changes from 0° to 45°. It was noticed that $U_{\mathrm{ped}}$ experienced an obvious increase, particularly at the Y-axis street and the windward side downstream of the X-axis street. $C_{\mathrm{ped}}^{*}$ decreased at the corresponding locations. However, a higher $C_{\mathrm{ped}}^{*}$ was present upstream of the X-axis street. Figure 7c,f shows the difference between $U_{\mathrm{ped}}$ and $C_{\mathrm{ped}}^{*}$ when φ changes from 0° to 90°. The X-axis experiences a higher $U_{\mathrm{ped}}$ with no obvious decrease in $U_{\mathrm{ped}}$ on the Y-axis street. The accumulation effect downstream of the X-axis street, which is narrower than the Y-axis street, leads to an obvious increase in $C_{\mathrm{ped}}^{*}$.

Figure 7g,j displays the $U_{\mathrm{ped}}$ and $C_{\mathrm{ped}}^{*}$ for the T-shaped intersection for the wind direction φ = 90°, that is, Case C-90. There exists a lower $U_{\mathrm{ped}}$ at both sides of the X-axis street, the intersection, and the right sides of the Y-axis street. Thus, a relatively higher $C_{\mathrm{ped}}^{*}$ was observed in the X-axis street and on the right side of the Y-axis street. Figure 7h,k displays the differences in $U_{\mathrm{ped}}$ and $C_{\mathrm{ped}}^{*}$ between Cases C-45 and C-90, the latter taken as the control case. It was noticed that $U_{\mathrm{ped}}$ experienced an overall increase, except for the upstream and central areas downstream of the Y-axis street. Therefore, $C_{\mathrm{ped}}^{*}$ decreased in these areas. Figure 7i,n displays the difference in $U_{\mathrm{ped}}$ and $C_{\mathrm{ped}}^{*}$ between Cases C-0 and C-90. Overall, the X-axis street experiences an increase in $U_{\mathrm{ped}}$, while the Y-axis street experiences the opposite. We observed that $C_{\mathrm{ped}}^{*}$ increased particularly on the left side of the Y-axis street.

**Figure 8:**
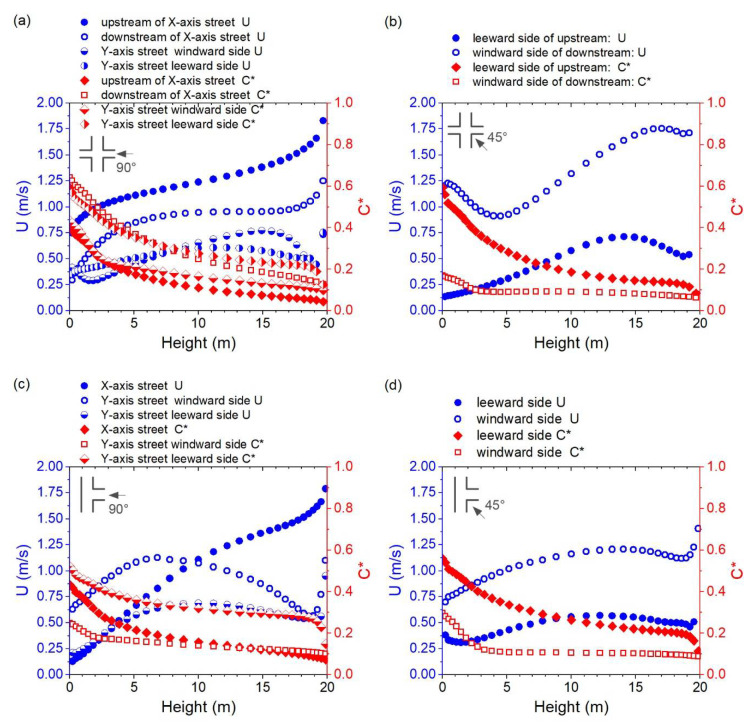
Vertical distribution of wind velocity (U) and normalized pollutant concentration (C*) for the near-wall surfaces in Cases (**a**) B-90, (**b**) B-45, (**c**) C-90, and (**d**) C-45.

Figure 8a,b displays the variation in wind velocity ($U_{\mathrm{nwz}}$) and normalized pollutant concentration ($C_{\mathrm{nwz}}^{*}$) along the height in the near-wall surface in Case B. For B-90, there was an obvious difference between the $U_{\mathrm{nwz}}$ for the upstream and downstream of the X-axis street. Therefore, $C_{\mathrm{nwz}}^{*}$ in the downstream region was distinguished from the upstream region. The $U_{\mathrm{nwz}}$ on the Y-axis street windward side was larger or less than the leeward side in different height ranges. However, the $C_{\mathrm{nwz}}^{*}$ on the windward side was generally lower, which indicates that the windward side near-wall zone has a greater pollutant removal ability. For Case B-45, the $U_{\mathrm{nwz}}$ on the leeward side, that is, the near-wall surfaces of the right-down building, was generally lower than on the windward side, that is, the near-wall surfaces of the left-up building. $U_{\mathrm{nwz}}$ reached its highest value at the upper middle of the canyon, and the $U_{\mathrm{nwz}}$ on the windward side showed a decreasing trend below 4 m. The $C_{\mathrm{nwz}}^{*}$ on the leeward side was generally higher, and it may achieve two or three times the $C_{\mathrm{nwz}}^{*}$ on the windward side, particularly at heights below 5 m.

Figure 8c,d displays the variation of $U_{\mathrm{nwz}}$ and $C_{\mathrm{nwz}}^{*}$ along the height in the near-wall surface in Case C. For Case C-90, the $U_{\mathrm{nwz}}$ in the X-axis street, the only inflow path, showed an increasing trend along the height, while $C_{\mathrm{nwz}}^{*}$ showed the opposite trend. The $U_{\mathrm{nwz}}$ on the Y-axis street windward side was obviously higher than that on the leeward side, and the highest value existed at the lower middle of the canyon, except for the velocity increasing near the top of the canyon. The $C_{\mathrm{nwz}}^{*}$ in the windward side was significantly lower, and it was lower than $C_{\mathrm{nwz}}^{*}$ in the Y-axis at heights below 8 m. For Case C-45, $U_{\mathrm{nwz}}$ showed a relatively slower changing trend along the height, except for the range near the top of the canyon. The $U_{\mathrm{nwz}}$ on the windward side was generally higher, and the $C_{\mathrm{nwz}}^{*}$ on the windward side was lower.

**Figure 9:**
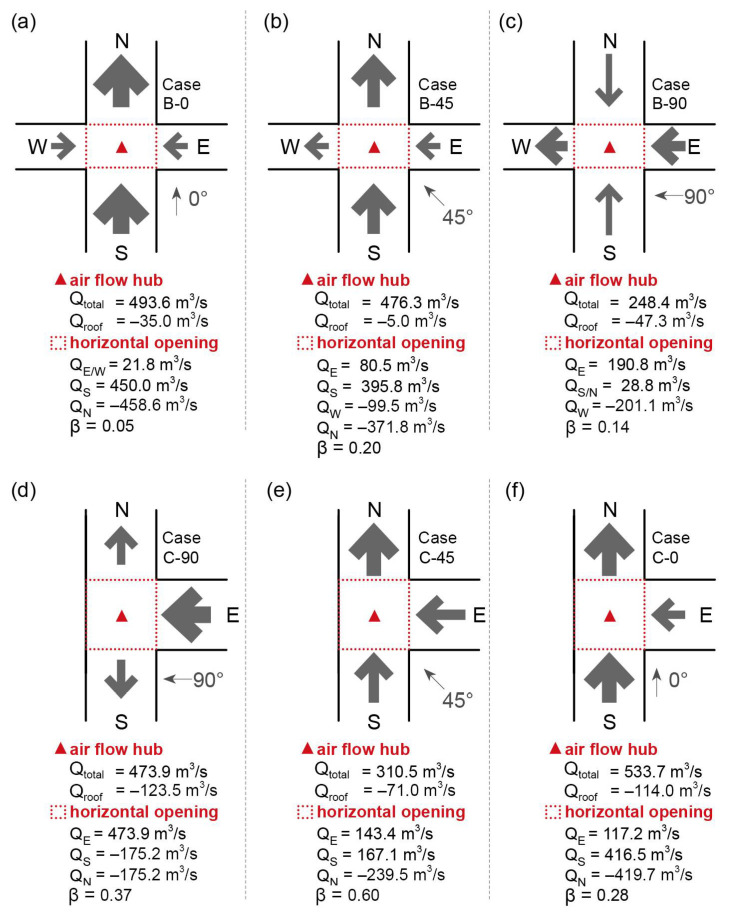
Volumetric flow rate for intersections and interfaces in Cases (**a**) B-0, (**b**) B-45, (**c**) B-90, (**d**) C-90, (**e**) C-45, and (**f**) C-0.

Figure 9a, Figure 9b, and Figure 9c show the flow rates for the intersections in Cases B-0, B-45, and B-90, respectively. The total volumetric flow rate (Q_total_) of the intersection in Case B-0 reached 493.6 m^3^/s. Similar to Case A-0 (Figure 6a), the south interface supplies a large proportion of inflow, in addition to the contribution from the east and west interfaces. An even smaller flow ratio (β) of 0.05 indicates less air exchange for the X-axis street. The outflow mainly passed through the northern interface. For Case B-45, a Q_total_ similar to that of Case B-0 was observed, and unbalanced ventilation was indicated by the β of 0.20. The narrower X-axis street showed a significantly lower air-exchange rate. For Case B-45, a much smaller Q_total_ was observed than for Cases B-0 and B-45. The east interface supplied a large proportion of the inflow owing to the strong channel effect. In addition, the vertical outflow at the intersection top opening accounts for approximately 19.0% of Q_total_, which reflects a considerable potential for pollutant removal.

Figure 9d, Figure 9e, and Figure 9f show the flow rates for the intersection in Cases C-90, B-45, and B-90, respectively. The total volumetric flow rate (Q_total_) of the intersection in Case C-90, under wind direction φ = 90°, reached 473.9 m^3^/s, which was totally induced from the east interface. The north and south adjacent street segments have the same outflow rate, and the top opening accounts for over 26.1% of Q_total_ because of the vortex and reverse flow near the left building. When φ changed to 45°, Q_total_ decreased significantly by approximately 34.5%. The inflow comes from the south and east interfaces, and the former has a relatively higher inflow rate. The flow ratio (β) of 0.60 indicates balanced ventilation for the intersection and adjacent street segments. When φ changes from 90° to 0°, Q_total_ increases with the Y-axis street as the main flow path. The east interface only provided approximately 22.0% of the inflow, which means there was unbalanced ventilation at the intersection.

Table 1 summarizes the spatially averaged $\bar{C^{*}}$ for the pedestrian zone (0–2 m), near-wall zone (within 1 m from the facade), and canopy layer (0–20 m), referred to as $\bar{C_{\mathrm{ped}}^{*}}$, $\bar{C_{\mathrm{nwz}}^{*}}$ and $\bar{C_{\mathrm{cnp}}^{*}}$, respectively, at three right-angled intersections. Detailed domains for the three target zones are shown in Figure 3 and discussed in Section 2.3. For Case A of “+”-shaped intersection, the $\bar{C_{\mathrm{ped}}^{*}}$ and $\bar{C_{\mathrm{cnp}}^{*}}$ showed similar decreasing extent when the wind direction (φ) change from 0° to 45°. However, the $\bar{C_{\mathrm{nwz}}^{*}}$ showed a less obvious change. For Case B of “+*”-shaped intersection, the $\bar{C_{\mathrm{ped}}^{*}}$ and $\bar{C_{\mathrm{cnp}}^{*}}$ showed similar decreasing extent of over −30.0% when φ changed from 0° to 45°. Meanwhile, a less decreasing extent of $\bar{C_{\mathrm{nwz}}^{*}}$ was observed. When φ changed from 0° to 90° (parallel to the narrower street), a significant increase in $\bar{C_{\mathrm{nwz}}^{*}}$ was observed, in comparison to the changes in $\bar{C_{\mathrm{ped}}^{*}}$ and $\bar{C_{\mathrm{cnp}}^{*}}$. Based on the above, it can be preliminarily concluded that the change in wind direction has a similar impact on the pollutant concentration for the pedestrian zone and canopy layer. For Case C of the T-shaped intersection, Case C-90 was taken as the control case, in which the inflow wind direction was parallel to the axis of symmetry. It should be noted that when φ changes from 90° to 0° or 270°, also perpendicular to the building arrays, the $\bar{C_{\mathrm{ped}}^{*}}$ showed a more significant increase, whereas when φ changed to oblique values, the $\bar{C_{\mathrm{cnp}}^{*}}$ showed a more significant decrease.

### 3.2. Ventilation and Pollutant Concentration at Oblique-Angled Intersections

**Figure 10:**
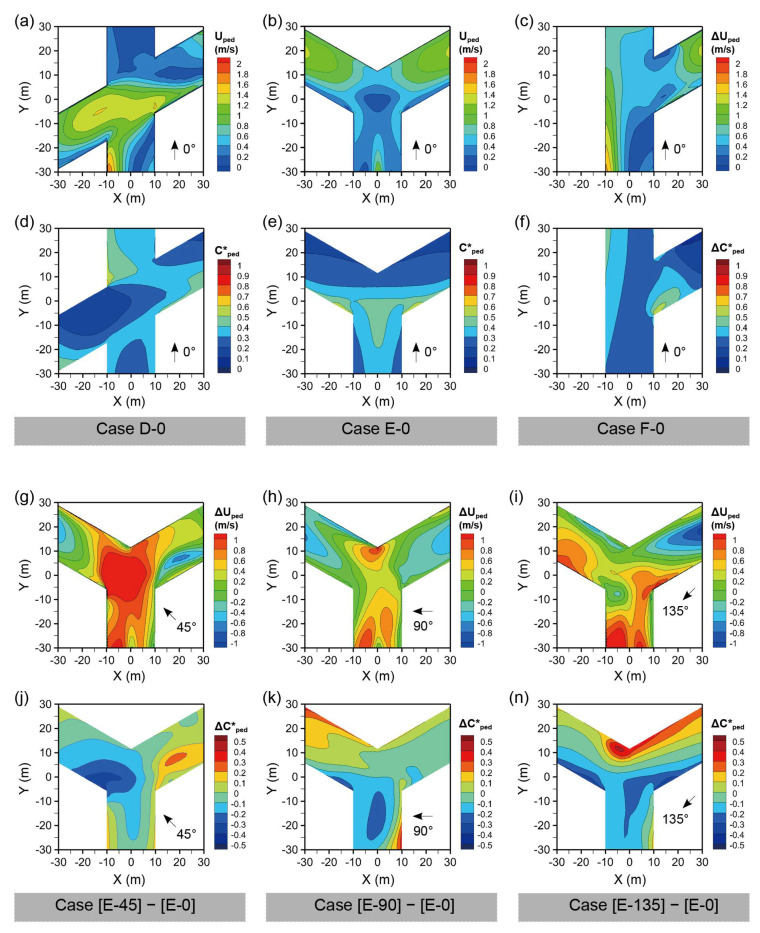
Wind velocity (U) and normalized pollutant concentration ($C^{*}$) at Z = 1.5 m for Cases (**a**,**d**) D-0, (**b**,**e**) E-0, and (**c**,**f**) F-0; the difference of wind velocity and normalized pollutant concentration for (**g**–**n**) Case E, taking Case E-0 as the control case.

Figure 10 shows the wind velocity ($U_{\mathrm{ped}}$) and normalized pollutant concentration ($C_{\mathrm{ped}}^{*}$) at the pedestrian level for the oblique-angled intersection cases, including Cases D (X-shaped), E (Y-shaped), and F (r-shaped).

Figure 10a–f shows the $U_{\mathrm{ped}}$ and $C_{\mathrm{ped}}^{*}$ for three oblique-angled intersections under wind direction φ = 0°. For Case D-0, there exists a larger $U_{\mathrm{ped}}$ at the left street segments, the intersection, and the left side of the down street segments, which indicates that the X-shaped intersection improves the pedestrian ventilation for upstream street segments. Therefore, more areas of the left and lower streets experienced lower $C_{\mathrm{ped}}^{*}$. For Case E-0, lower $U_{\mathrm{ped}}$ values were observed on both sides of the Y-axis street, leeward sides of the downstream street, and especially the central area of the intersection. In contrast, the downstream branches experience higher $U_{\mathrm{ped}}$ and lower $C_{\mathrm{ped}}^{*}$, particularly on the windward sides of buildings. For Case F-0, $U_{\mathrm{ped}}$ was lower at the intersection and on the right side of the Y-axis street. While, $C_{\mathrm{ped}}^{*}$ was higher on the left side of the Y-axis street and the leeward corner of the right-down building, which was caused by the reverse flow and local vortex.

Figure 10g–n displays the Δ$U_{\mathrm{ped}}$ and Δ$C_{\mathrm{ped}}^{*}$ for Cases E-45, E-90, and E-135, with Case E-0 as the control case. The φ of 45° contributed to an obvious increase in $U_{\mathrm{ped}}$ at the intersection and the Y-axis street, but a decrease in $U_{\mathrm{ped}}$ at the leeward side of the right-down building. Consequently, a decrease in $C_{\mathrm{ped}}^{*}$ was observed in the left and down street segments with smaller angles with the prevailing wind, while the right street experienced an increase in $C_{\mathrm{ped}}^{*}$, especially on the leeward side. The comparison between Cases E-90 and E-0 shows that the intersection and down street segments have increased $U_{\mathrm{ped}}$ and decreased $C_{\mathrm{ped}}^{*}$. However, downstream of the left street segment decreased $C_{\mathrm{ped}}^{*}$. For φ = 135°, the two downstream street segments experienced an increase in $U_{\mathrm{ped}}$ and a decrease in $C_{\mathrm{ped}}^{*}$. The upstream street, and especially the leeward sides of the building, experienced an obvious increase in $C_{\mathrm{ped}}^{*}$.

**Figure 11:**
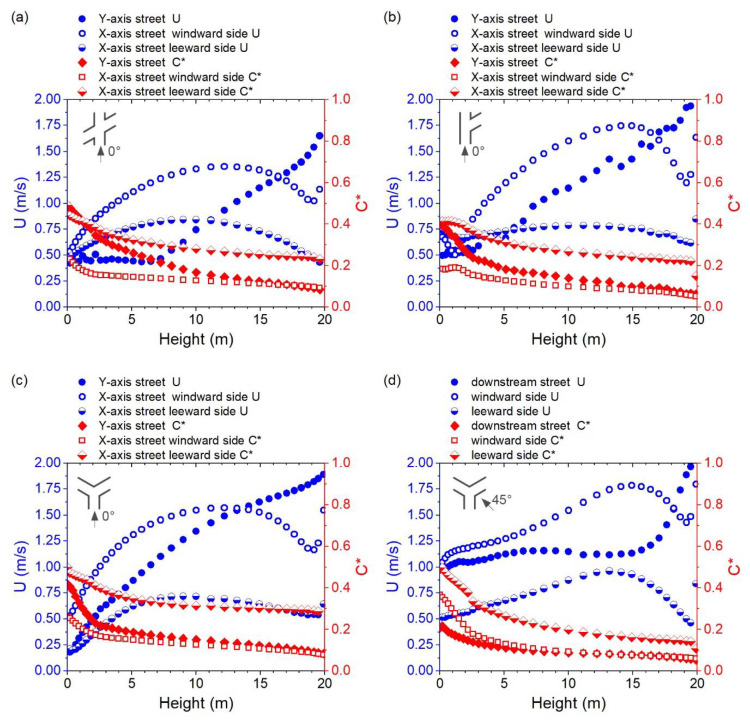
Vertical distribution of wind velocity (U) and normalized pollutant concentration (C*) for the near-wall surfaces in Cases (**a**) C-0, (**b**) F-0, (**c**) E-0, and (**d**) E-45.

Figure 11a–c shows the variation in wind velocity ($U_{\mathrm{nwz}}$) and normalized pollutant concentration ($C_{\mathrm{nwz}}^{*}$) along the height of the near-wall surface in Cases D-0, F-0, and E-0. The changing trends of $U_{\mathrm{nwz}}$ and $C_{\mathrm{nwz}}^{*}$ on both sides of the street parallel to the inflow wind direction, at the windward or leeward sides of the street, show similar changing trends along the height in these three cases, which was also found for Cases A-0, B-90, and C-90. Figure 11d shows the variation of $U_{\mathrm{nwz}}$ and $C_{\mathrm{nwz}}^{*}$ along the height in Case E-45. The changing gradient of $U_{\mathrm{nwz}}$ was generally lower, except for those above 15 m.

**Figure 12:**
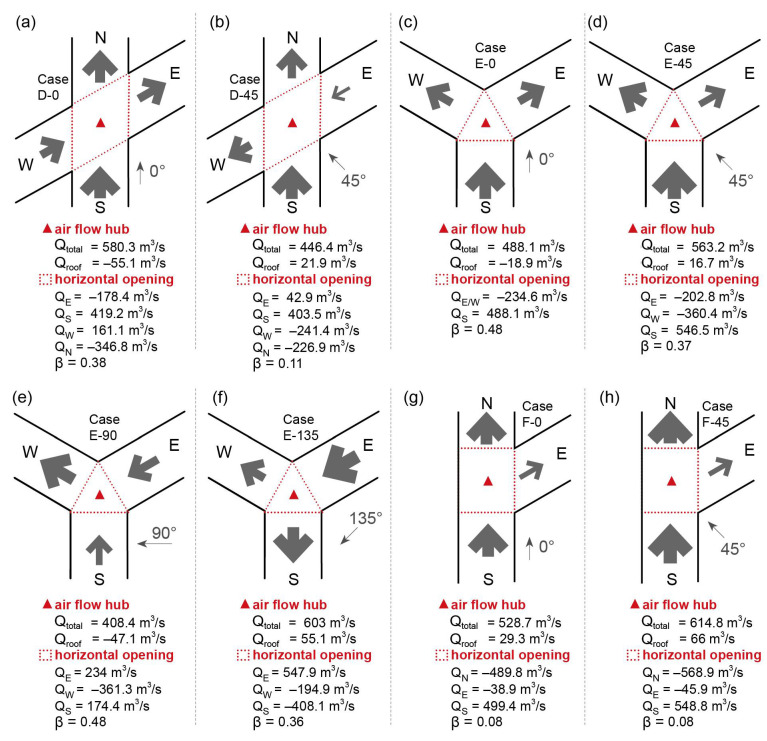
Volumetric flow rate for the intersection and its interfaces in Cases (**a**,**b**) D, (**c**–**f**) E, and (**g**,**h**) F.

Figure 12a,b displays the flow rate for the intersection in Cases D-0 and D-45. The intersection had a larger Q_total_ and a higher β value when the wind direction (φ) was parallel to the Y-axis. In contrast, the east street segment provided a limited inflow when φ = 45°. The main reason for this is the large angle between the east street orientation and the approaching wind direction. Figure 12c–f displays the flow rate for the intersection in Case E. The Q_total_ reached a maximum at 135°, with a small angle to the east street. Cases E-0 and E-90 have relatively higher values of β than those of Cases E-45 and E-135, in which there exists one street segment that has a significantly lower amount of air exchange with the intersection. Figure 12g,h displays the flow rate for the intersection in Cases F-0 and F-45. Both cases have quite low β values, which reflect only the Y-axis street serving as the main flow path, and there was a limited flow rate (≤100 m^3^/s) between the intersection and east street.

Table 2 summarizes the spatially averaged $\bar{C^{*}}$ values for the pedestrian zone, near-wall zone, and canopy layer at three oblique-angled intersections. For Case D of the X-shaped intersection, a significant decrease in $\bar{C_{\mathrm{nwz}}^{*}}$ was observed when the wind direction (φ) changed from 0° to 45°. When φ changed to 90° or 135°, there was no obvious change, all below 10%, of the spatially averaged $\bar{C^{*}}$. For Case E of the Y-shaped intersection, $\bar{C^{*}}$ decreased significantly, especially the $\bar{C_{\mathrm{cnp}}^{*}}$, when φ changed to 45°. The $\bar{C_{\mathrm{cnp}}^{*}}$, $\bar{C_{\mathrm{ped}}^{*}}$, and $\bar{C_{\mathrm{nwz}}^{*}}$ showed more obvious changes when φ changed to 90°, 135°, and 180°, respectively. A decrease in $\bar{C_{\mathrm{ped}}^{*}}$, and an increase in $\bar{C_{\mathrm{nwz}}^{*}}$ and $\bar{C_{\mathrm{cnp}}^{*}}$ were observed when comparing Cases E-180 and E-0. For Case F of the r-shaped intersection, $\bar{C_{\mathrm{ped}}^{*}}$ and $\bar{C_{\mathrm{cnp}}^{*}}$ showed a similar decrease when φ changed to 45°, 90°, 225°, and 315°. A more obvious increase in $\bar{C_{\mathrm{nwz}}^{*}}$ was observed when φ changed to 135° and 180°. There is an obvious increase in $\bar{C^{*}}$ for all three domains when comparing Case F-270 to F-0, which is mainly because there is no opening of the r-shaped intersection for φ = 270°.

### 3.3. Comprehensive Ventilation Analysis

To comprehensively evaluate the ventilation at different types of intersections, the overall flow conditions for each case under the given simulation inflow conditions were first compared, followed by an analysis of the divergent responses of $\bar{\tau_{\mathrm{ped}}^{*}}$, $\bar{\tau_{\mathrm{nwz}}^{*}}$, and $\bar{\tau_{\mathrm{cnp}}^{*}}$ to the change in intersection typology and approaching wind direction.

**Figure 13:**
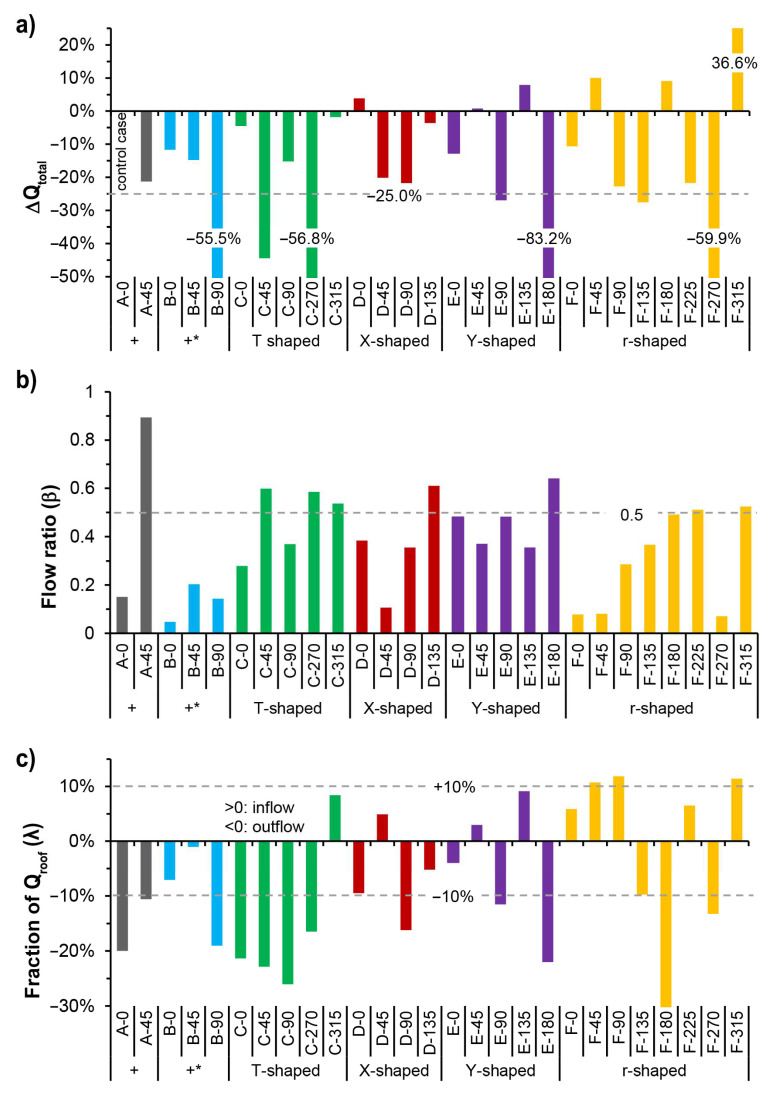
Flow rate conditions for all six types of intersections: (**a**) difference of Q_total_ compared to that of Case A-0; (**b**) flow ratio (β) for each case; (**c**) fraction of Q_roof_ (λ) for each case.

Figure 13a displays the difference of Q_total_ for all the cases compared to Case A-0, the “+”-shaped intersection under wind direction (φ) of 0°. It was observed that all the cases of right-angled intersections have a lower Q_total_, especially Case B-90 φ parallel to the narrower street, Case C-45 with φ 45°, oblique to the building array that led to a larger inflow cross-sectional area, and Case C-270 with no opening in the inflow direction. Meanwhile, the difference in Q_total_ between Cases C-0, C-315, and A-0 was within 5%. For the cases of oblique-angled intersections, larger differences may appear; for example, Cases E-180 and F-270 have significantly lower Q_total_, and Case F-315 has an obviously higher Q_total_. For Case D of the X-shaped intersection, Q_total_ was higher when φ was parallel to either the Y-axis or X-axis street. For Case E of the Y-shaped intersection, Q_total_ was higher under oblique φ angles of 45° and 135°. For Case F of the r-shaped asymmetrical intersection, Q_total_ was higher under oblique φ angles of 45°, 180°, and 315°.

Figure 13b displays the minimum flow ratio (β) between the horizontal openings at intersections for all cases. The β for Case A-45 reached the highest value of 0.89, which means that all four adjacent street canyons have similar air-exchange rates with the intersection area. The Case B of “+*”-shaped intersection has the lowest average value of β in all three groups of four-way intersection, which is due to the great difference of cross-sectional area between X-axis and Y-axis streets. For Case D of the X-shaped intersection, β was relatively higher, particularly under the approaching wind direction of 135°, which had a smaller angle of 15° with the oblique X-axis street. For other cases of three-way intersections, it was common for one of the adjacent street canyons to serve as the only inflow or outflow path. Thus, a considerably balanced ventilation can be expected when β reaches 0.5. It was noticed that the symmetrical cases, that is, Case C of the T-shaped intersection and Case E of the Y-shaped intersection, had a higher average value of β, compared to Case F of the asymmetrical r-shaped intersection. Subsequently, Cases F-0, F-45, and F-270 may experience significant differences in pollutant concentrations at different street canyons near the intersection.

Figure 13c displays the fraction of Q_roof_ to Q_total_ (λ) at the intersections for all cases. A positive λ means there exists an inflow to the intersection at the roof opening, while a negative λ indicates there is outflow from the intersection to the upper boundary layer. Case C of the T-shaped intersection had the largest average value of λ absolute values. The other cases normally have a value of λ approximately or less than ± 10%, except for Cases A-0, B-90, and F-180, in which the approaching wind direction (φ) was parallel to one of the streets, and Cases E-180 and F-270, in which there exists no opening in the inflow direction and thus leads to a considerable vertical air-exchange by the local vortex, and Cases D-90, E-90, and F-90, in which there exists an angle of 30° between φ and the inflow street, thus forming a three-dimensional swirling airflow at the intersection.

**Figure 14:**
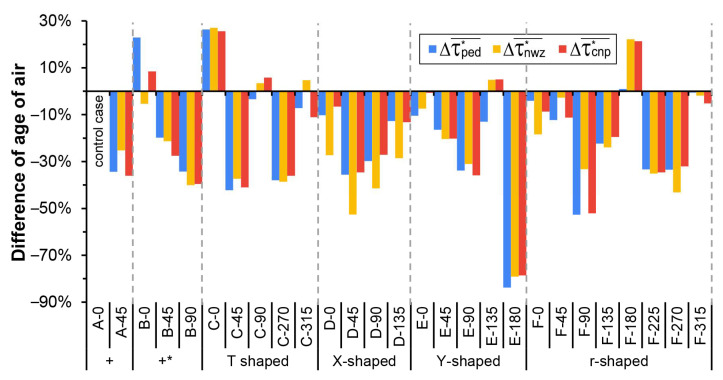
Difference of spatially average normalized age of air ($\bar{\tau^{*}}$) for all cases, taking Case A-0 as the control case.

The results shown in Figure 13c indicate that Q_total_ for the six groups of cases with different intersection typologies differ greatly under given inflow conditions, that is, the same wind velocity profile and different wind directions. Therefore, it should be noted that the spatially averaged pollutant concentration ($C^{*}$) shown in Table 1 and Table 2 was calculated without considering the difference between the flow rates at the intersections of different cases. To further explore the pollutant removal capacity of each case, the normalized indices of $\bar{\tau_{\mathrm{ped}}^{*}}$, $\bar{\tau_{\mathrm{nwz}}^{*}}$, and $\bar{\tau_{\mathrm{cnp}}^{*}}$ were calculated by considering the influence of the total volumetric flow rate (Q_total_) and volume of the studied area (V_cnp_). Figure 14 displays the difference in $\bar{\tau_{\mathrm{ped}}^{*}}$,$\;\bar{\tau_{\mathrm{nwz}}^{*}}$, and$\;\bar{\tau_{\mathrm{cnp}}^{*}}$ between Case A-0 and other cases. It can be observed that Cases A-45, B-90, C-45, C-270, D-45, D-90, E-90, E-180, F-90, F-225, and F-270 had overall lower $\bar{\tau^{*}}$. Specifically, $\bar{\tau_{\mathrm{ped}}^{*}}$, $\bar{\tau_{\mathrm{nwz}}^{*}}$, and $\bar{\tau_{\mathrm{cnp}}^{*}}\;$for Cases B-45, B-90, C-0, C-270, E-180, and F-225 showed similar changing trends. The differences in $\bar{\tau_{\mathrm{ped}}^{*}}$ and $\bar{\tau_{\mathrm{cnp}}^{*}}$ were similar for the above cases, while $\bar{\tau_{\mathrm{nwz}}^{*}}$ for Cases A-45 and F-90 showed relatively less variation, and $\bar{\tau_{\mathrm{nwz}}^{*}}$ for Cases D-45, D-90, and F-270 showed relatively greater changes. It was also noticed that $\bar{\tau_{\mathrm{ped}}^{*}}$ for Case B-0 showed a more significant increase compared to $\bar{\tau_{\mathrm{nwz}}^{*}}$, and $\bar{\tau_{\mathrm{cnp}}^{*}}$. For Cases D-0 and D-135, $\bar{\tau_{\mathrm{nwz}}^{*}}$ showed a relatively greater decrease. For Case F-180, $\bar{\tau_{\mathrm{nwz}}^{*}}$ and $\bar{\tau_{\mathrm{cnp}}^{*}}$ showed a significant increase compared with $\bar{\tau_{\mathrm{ped}}^{*}}$. These differences reflect the divergence in ventilation and pollutant diffusion of pedestrian zones, near-wall zones, and the canopy layer.

## 4. Limitations and Prospects

Although this study investigated only six types of intersections under homogeneous urban texture, it provides particular insight into the influence of intersection typology and wind direction on the ventilation and pollutant dispersion in pedestrian zones, near-wall zones, and the canopy layer. Other factors, such as the street length–width ratio [15], nearby building morphology [11,12], and surrounding urban context [33], have been confirmed to influence urban ventilation in previous studies, and should be further studied. The vehicle emissions were assumed to be uniform and evenly mixed at the bottom of the street canyon, that is, from the ground to 2 m, which is consistent with the setting of Hang et al. [24]. Different settings were seen in relevant studies [9,34], and it can be expected that the emission intensities of pollutants are affected to varying degrees owing to the different average traffic speeds at various intersections. Therefore, the pollution source settings should be further studied by employing experimental studies [35]. Under low wind speed conditions, solar radiation and convective heat transfer play leading roles in the ventilation of street canyons and intersections [36] and need to be further studied using non-isothermal simulations. Moreover, to further explore the impact of ventilation at intersections, pedestrian exposure to traffic-related pollutants, indices such as the personal intake fraction [14] and detailed modeling methods for sidewalks, trees, and moving vehicles [37], need to be considered. Notably, the pollutant concentration in the near-wall zone affects the indoor air quality in complex ways. For example, a wind flow parallel to windows brings significantly less air and pollutants into a room compared with a perpendicular flow [38]. As such, coupled outdoor and indoor air quality modeling approaches [14,39] should be employed to explore resident exposure, including outdoor air flow simulation and building ventilation that considers adaptive hybrid natural and mechanical ventilation strategies [40].

## 5. Conclusions

We studied the flow and pollutant dispersion of six intersection models with different typologies (right-angled and oblique-angled) for eight wind directions. The distribution and spatially averaged values of wind velocity and normalized pollutant concentration for the pedestrian zone, the near-wall zone, and the canopy layer were analyzed. The total volumetric flow rate (Q_total_), the minimum flow ratio (β) between the horizontal openings of the intersection, the ratio of Q_roof_ to Q_total_ (λ), and the spatially averaged normalized age of air ($\bar{\tau^{*}}$) were used to evaluate the ventilation at intersections. The main conclusions are summarized as follows:

- Right-angled intersections have a larger Q_total_ but lower β values because of the significant channel effect when the wind direction is parallel to the street canyon. Oblique wind directions led to a lower Q_total_ but a higher β value. For T-shaped intersections, a larger cross-sectional area for the outflow appears when the wind direction (φ) = 315°, which helps to increase Q_total_.
- Oblique-angled intersections experience a more significant difference in Q_total_ when the wind direction changes. However, β shows higher stability except for the r-shaped asymmetrical intersection or X-shaped intersection for φ = 45°, oblique to the building arrays.
- The vertical air-exchange rates for intersections are considerable when the wind directions are parallel to the street orientation, for example, “+”-shaped intersection when φ = 0°, or when there is no opening in the inflow direction, for example, a Y-shaped intersection when φ = 180°.
- For most cases, the $\bar{C^{*}}$ and $\bar{\tau^{*}}$ values for the pedestrian zone and the canopy layer showed similar changes to φ, compared to those for the near-wall zone. There are still cases in which only the $\bar{\tau_{\mathrm{ped}}^{*}}$ or $\bar{\tau_{\mathrm{nwz}}^{*}}$ changed substantially; for example, when the Y-shaped intersection experienced a change in the inflow wind direction from 0° to 135°. This reflects the divergence in ventilation and pollutant diffusion at different locations of the intersections.

This study provides insight into the divergent responses of the ventilation and pollutant concatenation of the pedestrian zone, the near-wall zone, and the canopy layer, to changes in intersection typology and wind direction. These results can be directly used to evaluate the environmental quality of intersections under specific prevailing wind directions. Present urban ventilation assessments need to pay closer attention to the risk of pollutant accumulation near building facades, as this impacts the amount of resident exposure to pollutants. In future research, the real neighborhood environment and overall performance of multiple intersections in an area should be further considered, which will help gain more practical knowledge for optimizing local urban design.

## Figures and Tables

**Table 1:** Spatially averaged normalized pollutant concentration for the pedestrian zone ($\bar{C_{\mathrm{ped}}^{*}}$), the near-wall zone ($\bar{C_{\mathrm{nwz}}^{*}}$), and the canopy layer ($\bar{C_{\mathrm{cnp}}^{*}}$) at right-angled intersections.

Cases		$\bar{C_{\mathrm{ped}}^{*}}$	$\bar{C_{\mathrm{nwz}}^{*}}$	$\bar{C_{\mathrm{cnp}}^{*}}$
**Case A** (“**+**”-shaped)	**A-0**	**0.37**	**0.24**	**0.22**
	A-45	0.31 (−16.6%)	0.22 (−5.1%)	0.18 (−18.8%)
**Case B** (“+*”-shaped)	**B-0**	**0.42**	**0.20**	**0.22**
	B-45	0.28 (−32.4%)	0.18 (−13.8%)	0.15 (−30.7%)
	B-90	0.44 (6.3%)	0.26 (25.9%)	0.25 (10.8%)
**Case C** (T-shaped)	**C-90**	**0.34**	**0.23**	**0.22**
	C-0	0.39 (16.1%)	0.25 (9.0%)	0.24 (5.5%)
	C-45	0.31 (−8.7%)	0.21 (−7.5%)	0.19 (−14.8%)
	C-270	0.43 (26.1%)	0.27 (16.4%)	0.27 (18.6%)
	C-315	0.28 (−17.0%)	0.20 (−12.6%)	0.16 (−27.4%)

Note. The results for the control cases, that is, Cases A-0, B-0, and C-90, of each group are in bold. The percentage differences of concentration are displayed in brackets.

**Table 2:** Spatially averaged normalized pollutant concentration for the pedestrian zone ($\bar{C_{\mathrm{ped}}^{*}}$), the near-wall zone ($\bar{C_{\mathrm{nwz}}^{*}}$), and the canopy layer ($\bar{C_{\mathrm{cnp}}^{*}}$) at oblique-angled intersections.

Cases		$\bar{C_{\mathrm{ped}}^{*}}$	$\bar{C_{\mathrm{nwz}}^{*}}$	$\bar{C_{\mathrm{cnp}}^{*}}$
**Case D** (X-shaped)	**D-0**	**0.34**	**0.21**	**0.21**
	D-45	0.32 (−6.7%)	0.17 (−15.3%)	0.20 (−9.0%)
	D-90	0.35 (3.8%)	0.22 (6.8%)	0.22 (3.5%)
	D-135	0.36 (4.8%)	0.22 (5.8%)	0.21 (0.0%)
**Case E** (Y-shaped)	**E-0**	**0.34**	**0.23**	**0.23**
	E-45	0.28 (−19.1%)	0.17 (−25.5%)	0.16 (−30.4%)
	E-90	0.30 (−11.7%)	0.20 (−10.9%)	0.18 (−22.9%)
	E-135	0.27 (−21.3%)	0.21 (−8.3%)	0.20 (−14.3%)
	E-180	0.32 (−6.0%)	0.27 (17.2%)	0.26 (11.8%)
**Case F** (r-shaped)	**F-0**	**0.33**	**0.18**	**0.19**
	F-45	0.25 (−25.7%)	0.17 (−3.0%)	0.15 (−21.0%)
	F-90	0.19 (−42.9%)	0.17 (−5.3%)	0.12 (−39.3%)
	F-135	0.33 (−0.1%)	0.21 (15.0%)	0.21 (8.6%)
	F-180	0.29 (−13.8%)	0.22 (22.7%)	0.21 (8.8%)
	F-225	0.26 (−20.7%)	0.16 (−9.2%)	0.16 (−18.3%)
	F-270	0.51 (54.7%)	0.28 (55.4%)	0.32 (65.9%)
	F-315	0.23 (−32.2%)	0.14 (−21.3%)	0.13 (−32.1%)

Note. The results for the control case, that is, Cases A-0, B-0, and C-90 (the inflow wind direction is parallel to the axis of symmetry), of each group is in bold. The percentage differences of concentration are displayed in brackets.
